# Occult hepatitis B virus infection: A major concern in HIV-infected patients: Occult HBV in HIV

**Published:** 2011-01-01

**Authors:** Amitis Ramezani, Mohammad Banifazl, Minoo Mohraz, Mehrnaz Rasoolinejad, Arezoo Aghakhani

**Affiliations:** 1 Department of Clinical Research, Pasteur Institute of Iran, Tehran, IR Iran; 2 Iranian Society for Support of Patients with Infectious Diseases, Tehran, IR Iran; 3 Iranian Research Center for HIV/AIDS, Tehran, IR Iran

**Keywords:** Human immunodeficiency virus, Hepatitis B virus, Hepatitis C virus

## Abstract

Human immunodeficiency virus (HIV)- infected patients are at risk of acquiring viral hepatitis, due to common routes of transmission. As the introduction of highly active antiretroviral therapy (HAART) reduced the frequency of opportunistic infections and improved survival, viral hepatitis emerged as an important cause of morbidity and mortality in HIV-infected cases. Occult hepatitis B virus (HBV) infection is characterized by presence of HBV infection without detectable hepatitis B surface antigen (HBsAg). There are conflicting reports on the impact of occult HBV infection on the natural history of HIV disease. In this review, we described the findings of studies on HIV and hepatitis B co-infection with focus on the prevalence of occult HBV infection. The results of this review demonstrated the importance of prevention, diagnosis and treatment of occult HBV infection in HIV-positive patients.

## Background

Hepatitis B virus (HBV) infection is a serious global health problem, with two billion people infected worldwide and 360 million suffering from chronic HBV infection [1]. HBV infection is the tenth leading cause of death worldwide, accounting for 520,000 to 1.2 million deaths each year [1][2][3]. The prevalence of chronic HBV infection varies considerably in different geographical regions. China, sub-Saharan Africa and the Amazon Basin, have the highest prevalence with chronic carrier rates more than 7%. Japan, the Indian subcontinent and southern parts of Eastern and Central Europe have intermediate prevalence with 2% to 7% of the population are chronic carriers of HBV. The Middle-East, Bahrain, Iran and Kuwait have low prevalence of chronic HBV infection (<2%) [2][4][5][6][7]. The prevalence of hepatitis B surface antigen (HBsAg) in Iran was reported to be between 2.5% and 7.2% in 1979 [8]. A more recent study showed that the prevalence of HBsAg is ranging from 1.7% to over 5% in different provinces [9]. Occult HBV infection (OBI) is characterized by presence of HBV infection without detectable HBsAg. Most occult HBV carriers have very low viremia levels so the diagnosis of OBI requires sensitive HBV-DNA PCR assay. A number of explanations for the persistence of HBV-DNA in HBsAg-negative samples have been proposed, including integration of HBV-DNA into host's chromosomes, mutations in the major hydrophilic loop of the S gene, the window period following acute HBV infection, underlying HCV co-infection, immunosuppression, poor ability of laboratory in detection of HBsAg and the presence of immune complexes in which HBsAg may be hidden [10][11][12][13]. The clinical implications of OBI involve different clinical aspects. OBI harbors potential risk for HBV transmission through hemodialysis, blood transfusion and organ transplantation. It can cause cryptogenic liver disease, acute exacerbation of chronic hepatitis B, or even fulminant hepatitis and development of hepatocellular carcinoma [10]. Human immunodeficiency virus (HIV) causes a chronic and latent infection in the body which induces extensive damage to the immune system. HIV-infected subjects show a quantitative depletion of CD4 cells and also an overall immune dysregulation [14]. HBV is a frequent co-contaminant with HIV, because both share common modes of transmission [14] and it is an important cause of mortality and morbidity among HIV-infected persons [15]. In co-infected patients, the mortality rate is 19 times higher than in HIV mono-infected individuals [16]. The high rates of serological markers of HBV infection have been reported in HIV populations and can be as high as 68% [17][18][19][20]. Isolated hepatitis B core antibodies (anti-HBc) are predominant serological pattern in these patients [17][21] and can be associated with OBI [19][22]. OBI in other high risk groups such as hemodialysis patients was reviewed and published in Iran [23]. In this review, we described the findings of studies on HIV and HBV co-infection with focus on the prevalence of OBI.

## Hepatitis B Virus Infection in HIV-Infected Patients

HIV- infected patients are at risk of acquiring parenterally- or sexually-transmitted viruses including HBV. Approximately 20% of HIV-positive patients who acquire acute HBV infection develop chronic HBV infection compared to only 5% of HIV-negative persons [15]. Chronic HBV infection affects 7%-10% of HIV-infected patients with large differences according to geographical region [7]. After the introduction of highly active antiretroviral therapy (HAART) liver disease has emerged as a major cause of morbidity and mortality in HIV-infected subjects [24]. In HIV-HBV co-infected patients, it has been suggested that HIV interferes with the natural history of HBV infection by enhancing HBV replication and higher HBV-DNA levels and decreased hepatitis B e-antigen (HBeAg) seroconversion, leading to more severe liver disease, more rapid progression of liver fibrosis and a higher rate of cirrhosis decompensation, end-stage liver disease and hepatocellular carcinoma [25][26]. There is controversial data on the activity of inflammatory liver disease in HBV-HIV co-infected patients. Some studies in Californian and French HIV-positive subjects showed increased necro-inflammatory activity [27][28]. In contrast, other studies from northern Europe and USA showed a significantly less severe necro-inflammatory activity in HIV-HBV co-infected men who have sex with men (MSM) [29][30]. Besides, one study from the Central African Republic identified a lower prevalence of chronic hepatitis B in HIV-infected vs HIV-uninfected individuals [31]. On the other hand, studies from the Cote d'Ivoire, Malawi, Thailand and Tanzania have reported similar chronic hepatitis B prevalence between HIV-uninfected and HIV-infected subjects [32][33][34][35].

## OBI in HIV-Infected Patients

The prevalence of OBI in HIV-positive patients varies considerably from 0% to 89.5% in different geographical regions [17][18][19][20][36]. These discrepancies in the rate of OBI may reflect the diverse prevalence of HBV and HIV infections in different countries and the sensitivity of the assays used to detect the HBV-DNA. OBI is most frequently seen in patients with anti-HBc antibody as the only HBV serological marker. However, no anti-HBc or anti-HBs antibody could be detected in some individuals [37][38]. Nunez, et al, [18] did not detect HBV-DNA among 85 HIV-positive injection drug users and Bloquel, et al, [39] found OBI in 0.8% of French HIV-infected patients. In two studies from Brazil [40] and the Netherlands [41] the rate of OBI was reported to be 5% in HIV-infected patients. In two other studies in South Africa, almost 22% of HIV-infected people without HBsAg had detectable HBV-DNA [36][42]. In another study, in two cohorts of HIV-positive patients in UK, approximately 14% of patients had OBI [43]. Hofer, et al. [17] identified HBV-DNA in 89.5% of patients who had isolated anti-HBc antibody. The other study by Firnhaber, et al. [44] showed that 88.4% of HIV-positive cases with isolated anti-HBc antibody had OBI. In another study in Iranian HIV-positive patients with isolated anti-HBc antibody, HBV-DNA was detected in 13.6% of the patients [25] compared to 24.5% of Indian and 28.7% of Lebanese HIV-infected patients with isolated anti-HBc [14][45].

## OBI in HIV-HCV Co-Infected Patients

Thirty-five million people have HIV infection worldwide, and approximately 20% of them had chronic hepatitis C (HCV) infection [46]. HCV infection affects 25% of HIV-positive subjects, with greater rates (approximately 75%) in persons acquired infection through contaminated blood or blood products and intravenous drug users. Some studies have demonstrated that HIV infection modifies the natural history of HCV infections, increasing the progression to chronic disease and cirrhosis [25]. With the advent of HAART, there has been a significant decrease in opportunistic infections, so HCV has emerged as an important cause of morbidity and mortality in HIV-infected patients. A study in French HIV-infected patients showed that deaths due to HCV-related end-stage liver disease were more frequent in the HAART era than in the pre-HAART era [47]. It was shown that HCV-HBV co-infection may result in a decrease in the replication of both viruses with greater effect on HBV. HCV core protein binds to HBV-DNA and suppresses HBV gene expression and replication [48]. OBI may be encountered in HIV-HCV co-infected patients and may cause more severe liver disease and lower response to interferon therapy [49]. Previous studies on the association between HCV infection and OBI persistence in HIV-infected cases have given conflicting results [17][18][21]. The percentage of OBI in HIV-HCV-positive patients varies considerably in different studies from less than 1% [50], to 1.4%-5% [51][52][53], 10% [54], 33%-35% [20][42] and more than 40% [55]. In another study 21% of HIV-HCV co-infected patients had detectable HBV-DNA in their plasma and OBI was significantly higher in HCV co-infected subjects than HCV-negative cases [56]. Laguno, et al. found that OBI presents in at least 6.3% of HCV-HIV patients and in more than 16% of cases with anti-HBc [57]. Fabris, et al. showed that 13.4% of HCV-HIV co-infected patients had OBI [58]. In another study in Iran HBV-DNA was detectable in 16.7% of HCV-HIV co-infected patients with isolated anti-HBc [59]. Despite the reports regarding to strong association between HCV infection and OBI [58][60][61][62], Jardim, et al. [40] reported no significant difference in the presence of HBV-DNA in HIV-positive patients with or without HCV infection. Daza, et al. [63] also found a very low rate of HBV-DNA in HIV-HCV co-infected patients. They reported that OBI is a rare finding in these patients.

## Conclusions

Hepatic abnormalities are common in HIV-infected subjects. Due to shared routes of transmission, chronic HBV and HCV infections frequently complicate HIV disease. To date, there are controversial evidences regarding the influence of HBV infection on HIV disease progression. While some early studies suggested that HBV-related liver disease may be less severe among HIV-infected patients, recent studies indicate an increased risk of liver-related morbidity and mortality among HIV-HBV co-infected patients. Current evidences demonstrated that OBI is relatively frequent in HIV-infected individuals. These data emphasize the importance of prevention, diagnosis and treatment of OBI in HIV-positive cases and all HIV-infected patients should be screened for evidence of resolved or active HBV infection. HCV infection is also common in HIV-infected individuals with parenteral exposure such as IDUs and recipients of blood products. Although the majority of HCV-HIV co-infected cases are asymptomatic, HCV infection may lead to the development of hepatic fibrosis, cirrhosis and hepatocellular carcinoma. There are conflicting reports on the impact of HCV infection on the natural history of HIV disease. With the advent of HAART, there has been a significant decrease in opportunistic infections so HCV has emerged as an important cause of morbidity and mortality in HIV-infected patients. Taken together, these data provide evidences of the increasing importance of HCV among HIV-infected subjects. There is limited information on the clinical impact and management of HIV-infected patients with OBI. Further studies to provide sufficient data for management of the clinical consequences of these patients are suggested.
